# Insulin resistance and its association with coronary heart disease: insights from bioinformatics analysis

**DOI:** 10.3389/fendo.2025.1730801

**Published:** 2025-12-12

**Authors:** Xinyue Lu, Minghuan Liu, Yang Song, Shiying Lin, Xiaowan Zhou, Hang Zeng, Aina Li, Huangyuan Li, Xiaoxu Xie, Shaowei Lin, Siying Wu

**Affiliations:** 1 Department of Epidemiology and Health Statistics, The School of Public Health, Fujian Medical University, Fuzhou, China; 2 Department of Cardiology, The First Affiliated Hospital of Fujian Medical University, Fuzhou, China; 3 Department of Preventive Medicine, The School of Public Health, Fujian Medical University, Fuzhou, China

**Keywords:** coronary heart disease, insulin resistance, fasting plasma glucose, bioinformatic enrichment analyses, mediating effect

## Abstract

**Background:**

Evidence on the relationship between no-insulin-based insulin resistance (IR) surrogates and the prevalence of coronary heart disease (CHD) in remains limited. Here, we assess the associations between multiple non–insulin-based IR surrogates and CHD, delineating subgroups at heightened susceptibility across demographic and cardiometabolic strata. We further explore plausible mechanistic pathways and perform mediation analyses to elucidate the pathophysiological links between IR and CHD.

**Methods:**

This study analyzed 7,419 participants from Fujian Medical University affiliated hospital (2018–2024). Multivariate logistic regression and restricted cubic splines (RCS) were performed to evaluate the relationship between the atherogenic index of plasma (AIP index), triglyceride glucose index (TyG index), the metabolic score for insulin resistance (METS-IR), and triglyceride(TG)/high density lipoprotein cholesterol(HDL-C) ratio (TG/HDL-C ratio) and the prevalence of CHD. Subgroup analysis and interaction analysis were employed to identify effect modifiers associated with the IR-CHD relationship, whilst bioinformatics analysis and mediation analysis were utilized to identify and quantify potential mediating effects within this association.

**Results:**

In primary analyses, surrogate markers of IR were positively associated with the prevalence of CHD, with odd ratios ranging between 1.039 and 1.612. Moreover, physical inactivity was the predominant effect modifier (interaction *P*-value < 0.05), with a possible additional influence of younger age, so that sedentary adults <60 years with elevated IR surrogates appeared particularly vulnerable. Mechanistically, bioinformatic enrichment analyses highlighted three core pathways, including AGE–RAGE signaling in diabetic complications, FoxO signaling, and adipocytokine signaling, providing context for the observed patterns. Finally, mediation analyses indicated that fasting plasma glucose mediated 8.15%–55.33% of the associations, including AIP index, TyG index, METS-IR, and ln(TG/HDL-C).

**Conclusion:**

In this large hospital-based cross-sectional sample, non–insulin-based IR surrogates were positively associated with the prevalence of CHD and may help identify groups with a higher likelihood of having CHD. Glycemic pathways and physical inactivity emerged as important correlates of these associations and deserve further evaluation in longitudinal studies.

## Graphical Abstract

**Figure:**
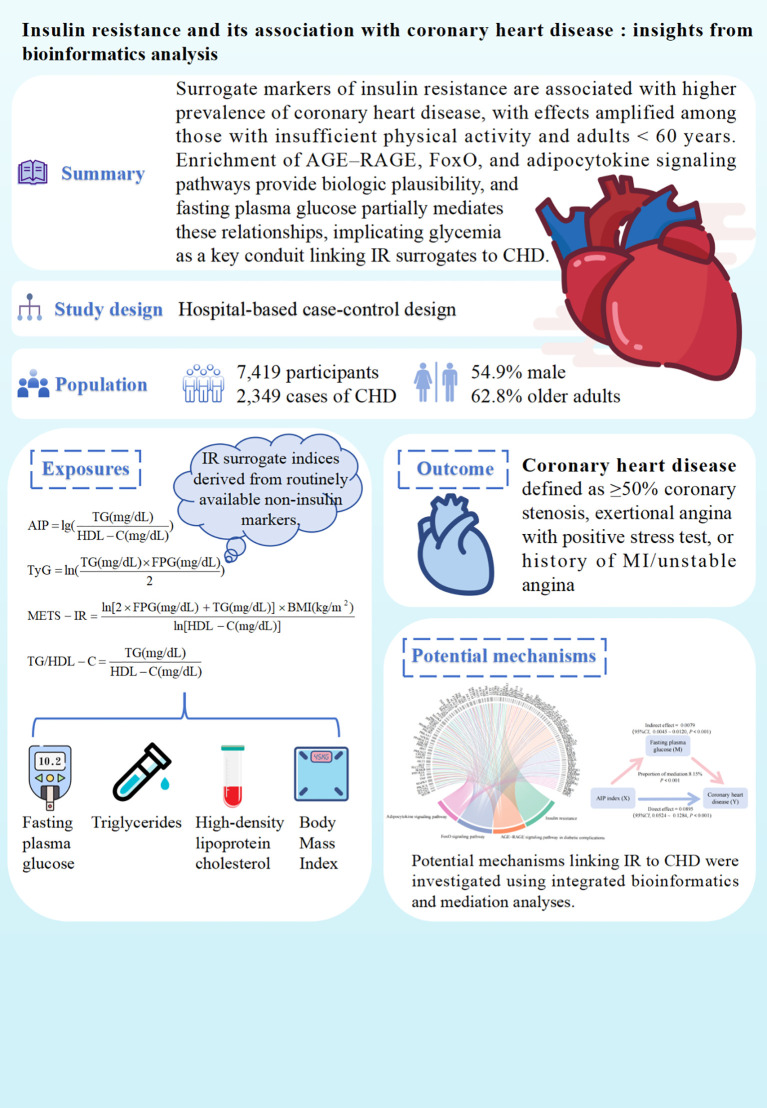

## 1 Introduction

Coronary heart disease (CHD) refers to the process of plaque formation within the major arteries supplying the myocardium, leading to reduced blood flow. Plaque calcification and rupture can lead to thrombosis, thereby posing life-threatening risks (1). It represents the most prevalent cardiovascular disease and is one of the leading causes of chronic disease-related mortality globally (2). Studies have demonstrated insulin resistance (IR) goes hand in hand with the extension and severity of coronary atherosclerosis in non-diabetic patients (3).

IR is characterized by metabolic dysregulation, particularly in glucose and lipid metabolism, which contributes to the development of CHD (4). Consequently, the early identification of IR prior to CHD onset is crucial. Currently, the hyperinsulinemic-euglycemic clamp (HIEC) remains the “gold standard” for assessing IR. However, HIEC is a complex, invasive technique that is time-consuming, labor-intensive, costly, and requires highly skilled operators to address a series of technical challenges (5). In recent years, novel composite indices integrating lipid parameters have emerged as alternative markers for evaluating IR, demonstrating superior predictive efficacy for metabolic syndrome and type 2 diabetes compared to single parameters. For instance, the atherogenic index of plasma (AIP), the TyG index, the metabolic score for insulin resistance (METS-IR), and triglyceride(TG)/high density lipoprotein cholesterol(HDL-C) ratio has been validated to correlate closely with HOMA-IR values and offers greater simplicity than HOMA-IR (6–8). Emerging evidence has established robust associations between these novel composite indices and cardiac metabolic disorders risk across diverse populations (9–11). Observational and longitudinal studies have reported that higher TyG, METS-IR, AIP and TG/HDL-C are associated with incident cardiovascular events, CHD or coronary atherosclerosis severity in different populations (12–16). However, data directly comparing multiple no-insulin-based IR surrogates in relation to CHD in Chinese remain limited.

Despite this, extant studies have chiefly concentrated on the correlation between IR and cardiometabolic diseases, yet overlooked the fundamental mechanisms underpinning this association. Fasting plasma glucose (FPG) as a key metabolic marker of IR, may mediate the association between IR and cardiovascular disease (17). IR can induce an imbalance in glucose metabolism that generates chronic hyperglycemia, which in turn triggers oxidative stress and causes an inflammatory response that leads to accelerating the formation of atherosclerotic plaques (18, 19). Clinical studies have confirmed that even in individuals with normal glucose tolerance, elevated FPG is independently associated with the severity of coronary artery lesions (20). Experimental models demonstrate that elevated FPG under IR conditions alters myocardial fatty acid utilization, shifting energy substrates towards monounsaturated fatty acids while reducing myocardial omega-3 polyunsaturated fatty acid content (21). The extant research outlined above lends support to the hypothesis that FPG may act as a potential mediating factor in the relationship between IR and CHD.

The objective of this cross-sectional study was to use hospital-based data from southeastern China to add to the existing evidence on IR surrogates and CHD. The following objectives were set out: firstly, to estimate the correlation between four IR surrogate indicators (AIP index, TyG index, METS-IR, and TG/HDL-C ratio) and CHD; secondly, to explore potential modifying factors of the association, given the complex, multifactorial nature of CHD; and thirdly, to identify and quantify the mediating proportion of FPG in this association through bioinformatics and mediation analysis, respectively.

## 2 Materials and methods

### 2.1 Study population

The preliminary screening phase of this study involved inpatients admitted to the Department of Cardiology at The First Affiliated Hospital of Fujian Medical University, a tertiary-level medical facility located in the southeastern region of Fujian Province, as well as individuals undergoing health examinations at the Union Hospital Affiliated to Fujian Medical University during the period spanning from January 2018 to March 2024. Researchers collected demographic data (e.g. gender, age, height, weight) and lifestyle information (e.g. smoking, drinking, physical activity). Following the exclusion of participants with missing diagnoses (84 cases), missing exposure variables (1,139 cases), or abnormal exposure measurements (330 cases), final 7,419 subjects were enrolled (**Supplementary Data Sheet 1****).** All participants or their legal representatives provided written informed consent. Prior to data extraction, the authors of the study anonymized the patient information, in accordance with the principles of the Declaration of Helsinki. The present study was approved by the Ethics Committee of the First Affiliated Hospital of Fujian Medical University.

### 2.2 Assessment of CHD

CHD was defined as having at least one of the following conditions: (1) percutaneous coronary angiography or computed tomographic angiography (CTA) examination showed that at least one coronary artery trunk or primary branch had ≥ 50% stenosis; (2) typical exertional angina symptoms with positive stress test; (3) previously diagnosed MI; (4) previously diagnosed unstable angina pectoris (22).

### 2.3 Definition of IR surrogate markers

Venous blood samples were collected from participants who had fasted for a minimum of 8h, including FPG, TG, HDL-C. The IR surrogates in this study include the AIP index, TyG index, and METS-IR and TG/HDL-C ratio (Equations 1–4). These indexes were calculated as formulas below (11, 23–25):

(1) $$\text{AIP}=\mathrm{lg}(\frac{\text{TG}\left(\text{mg}/\text{dL}\right)}{\text{HDL}-\text{C}\left(\text{mg}/\text{dL}\right)})$$

(23)

(2) $$\text{TyG}=\ln(\frac{\text{TG}\left(\text{mg}/\text{dL}\right)\times \text{FPG}\left(\text{mg}/\text{dL}\right)}{2})$$

(24)

(3) $$\text{METS}-\text{IR}=\frac{\ln\left[2\times \text{FPG}\left(\text{mg}/\text{dL}\right)+\text{TG}\left(\text{mg}/\text{dL}\right)\right]\times \text{BMI}\left(\text{kg}/{\text{m}}^{2}\right)}{\ln\left[\text{HDL}-\text{C}\left(\text{mg}/\text{dL}\right)\right]}$$

(11)

(4) $$\text{TG}/\text{HDL}-\text{C}=\frac{\text{TG}\left(\text{mg}/\text{dL}\right)}{\text{HDL}-\text{C}\left(\text{mg}/\text{dL}\right)}$$

(25)

### 2.4 Covariates and mediators

A series of covariates included as categorical or continuous variables in the analysis were as follow: demographic factors [gender (male or female), age (categorized as ≥ 60 or < 60 years), and body mass index (BMI)] and lifestyle factors [smoking status (never, current, or former), drinking (no or yes), physical activity (no or yes), and medical history (hypertension, hyperlipidemia). Specific coding details refer to **Supplementary Data Sheet 1**. FPG were considered as possible mediators in our mediation analysis.

The BMI was calculated using the formula: BMI (kg/m^2^) = body mass (kg)/height^2^(m^2^) (26). Diabetes mellitus was defined by FPG ≥ 7.0 mmol/L (126 mg/dL), a 2-h serum glucose level ≥ 11.1 mmol/L on oral glucose tolerance testing, or the current use of hypoglycemic drugs or insulin (27). The criteria for diagnosis of hypertension are Systolic Blood Pressure (SBP) ≥ 140 mmHg, diastolic blood pressure (DBP)  ≥  90 mmHg, self-reported specialist‐diagnosis history, or the use of antihypertensive medication (28). Hyperlipidemia was defined by a fasting serum total cholesterol level of more than 5.72 mmol/L (221 mg/dL) or triglyceride level of more than 1.7 mmol/L (150 mg/dL) (27). Physical activity cutoff value: Total Physical Activity MET minutes per week is < 600, that not meeting recommendations (29).

### 2.5 Identification and functional enrichment analysis of shared targets between IR and CHD

Gene targets related to insulin resistance and CHD were identified using the GeneCards (https://www.genecards.org) and OMIM (https://omim.org/) databases. We searched both databases with the keywords “insulin resistance” and “coronary heart disease”, restricting the species to Homo sapiens; all searches were performed on 31 May 2025. From GeneCards, we extracted genes together with their relevance “Score” and applied a median-based filter separately for each condition: genes with a Score ≥ 2.43 for “insulin resistance” and ≥ 6.77 for “coronary heart disease” were retained. OMIM entries corresponding to human genes were included using the same keyword terms without additional numeric thresholds. The resulting gene sets for insulin resistance and CHD were merged across GeneCards and OMIM, and their intersection was obtained using a Venn-diagram analysis. These overlapping genes were considered core candidate targets linking IR and CHD. The complete gene lists and the overlapping set are provided as files in the **Supplementary Data Sheet 2**.

To elucidate the biological functions of these overlapping targets, we conducted a comprehensive Gene Ontology (GO) enrichment analysis, encompassing biological processes (BP), cellular components (CC), and molecular functions (MF). This approach allowed for a systematic interpretation of the biological roles underlying the shared targets. In addition, Kyoto Encyclopedia of Genes and Genomes (KEGG) pathway enrichment analysis was performed to identify critical signaling pathways through which IR may influence the development and progression of CHD. Through these integrative analyses, we aimed to provide a more precise and mechanistic understanding of the molecular interactions bridging IR and CHD.

### 2.6 Statistical analysis

The results are expressed as median (interquartile range [*IQR*]) for continuous variables, and categorical variables are presented as numbers (percentages). We treated IR surrogates as continuous variables or categorical variables (tertile interval) in the analysis. Characteristics of the participants of the study are presented as descriptive statistics stratified by CHD status. The differences among these groups were examined using the Mann-Whitney U test for continuous variables and the Chi-square test for categorical variables.

For covariates with under 10% missing data, we used multiple imputation by chained equations (MICE) under a missing-at-random assumption, implemented in the mice package (version 3.18.0) to create five imputed datasets. The imputation model included covariates: smoking, drinking, metabolic equivalent of task, systolic and diastolic blood pressure, and total cholesterol. The standard trace plots of chain-specific showed good mixing and stable trajectories over iterations, without systematic trends, indicating satisfactory convergence of the MICE algorithm. Further details of the imputation procedure and convergence diagnostics are provided in the **Supplementary Data Sheet 1**.

We further used logistic regression models to explore the relationship between insulin resistance indices and CHD. IR indices were expressed as a continuous variable (per one-unit increase and per tertile increment) and a categorical variable (tertile) across three sequential adjustment levels: unadjusted model1, basic adjustment model 2 (adjusted gender and age), and full adjustment model 3 (adjusted BMI, drinking, smoking, physical activity, hypertension, hyperlipidemia on the basis of model 2). Model 3 was designated as the primary Model for all substantial inferences.

To explore the exposure-response relationship and to test for linearity between IR indices levels and CHD, we plotted restricted cubic spline curves with 3 knots at the 10th, 50th and 90th percentiles with the ‘rms’ and ‘splines’ packages in R software. Moreover, subgroup and interaction analyses were conducted to investigate potential heterogeneity in the association between the insulin resistance indices and CHD.

For the enrichment analysis, GO and KEGG terms with *P* values less than 0.05 were initially selected for inclusion. Thereafter, statistically significant terms were identified based on a threshold of false discovery rate (FDR) < 0.05, to control for multiple comparisons and reduce the likelihood of type I errors.

To further explore the potential mediating roles of insulin resistance indicators in the pathway linking IR to CHD, multivariate causal mediation analyses were conducted using the mediation package in R. A logistic regression framework was adopted due to the binary nature of the outcome variable. Specifically, the mediation models assessed the role of FPG as a mediator in the relationship between four insulin resistance indices and CHD risk. Within this framework, the Average Direct Effect (ADE) represents the proportion of the total effect that is independent of the mediator, whereas the Average Causal Mediation Effect (ACME) quantifies the effect mediated through FPG. The mediation proportion was calculated using the formula: ACME/(ADE + ACME) × 100%, indicating the extent to which the mediator accounts for the total effect. The parameters for mediation analysis are detailed in the **Supplementary Data Sheet 1**.

Additionally, we carried out several sensitivity analyses to confirm the stability of the main results. Firstly, in order to observe the stability of the results of the imputation-free dataset, this study conducted a correlation analysis on the imputation-free dataset. Second, to account for potential confounding by the sampling frame, the source of participants was further adjusted on the basis of the fully adjusted Model 3. We then evaluated effect modification by adding multiplicative interaction terms between each IR index and recruitment source (index × source) to the pooled models and deriving *P*-values for interaction. Third, the population is re-analyzed by inverse probability weighting with weights truncated at the 1st and 99th percentiles, and the result remains unchanged. Fourthly, given the impact of diabetic to CHD, these participants were excluded to. In the last, external random subsampling was conducted under the Probit link function (500 iterations; with 80% of the original sample being randomly sampled each time). The mediating effect was consistent with the primary analysis based on the Logit model.

All analyses were conducted using R v.4.5.0 or GraphPad Prism v.8.4, unless otherwise specified. A two-sided *P* < 0.05 was considered statistically significant.

## 3 Results

### 3.1 Baseline characteristics of participants

**Table 1** provides a concise overview of the characteristics of the study population. The total number of participants included in the study was 7,419, comprising 2,349 cases of CHD (31.7%). The demographic profile of the participants revealed a preponderance of males, constituting 54.9% of the sample. A significant proportion of the participants belonged to the elderly demographic, accounting for 62.8% of the sample, with ages 60 years and above constituting the majority. Statistically significant differences were identified between the two subgroups in most baseline characteristics, including gender, age, smoking, physical activity levels, and the prevalence of hypertension, diabetes mellitus, and hyperlipidemia. In addition, the median values for four insulin resistance surrogate markers (AIP index, TyG index, METS-IR, TG/HDL-C ratio) and FPG were higher in the case group than in the control group. Furthermore, statistically significant differences were also observed between subgroups (*P* < 0.05). Moreover, **Supplementary Data Sheet 1** showed the number of patients from the cardiology inpatient department and the health examination center were 4,788 and 2,631 respectively. several demographic and cardiometabolic variables differed significantly between the two groups (*P* < 0.05).

**Table 1:** Descriptive statistics of the study population.

Characteristics	Overall (n = 7,419)	Non-CHD (n = 5,070)	CHD (n = 2,349)	P value
AIP index*	0.39 (0.19, 0.58)	0.38 (0.18, 0.56)	0.42 (0.23, 0.61)	< 0.001
TyG index*	8.50 (8.14, 8.88)	8.48 (8.13, 8.87)	8.53 (8.16, 8.91)	0.006
TG/HDL-C ratio*	2.46 (1.55, 3.83)	2.39 (1.50, 3.67)	2.63 (1.68, 4.04)	< 0.001
METS-IR*	35.90 (31.43, 40.76)	35.29 (30.98, 40.20)	37.11 (32.51, 41.72)	< 0.001
Gender, n (%)				< 0.001
Male	4072 (54.9)	2509 (49.5)	1563 (66.5)	
Female	3347 (45.1)	2561 (50.5)	786 (33.5)	
Age, years, n (%)				< 0.001
< 60	2761 (37.2)	2191 (43.2)	570 (24.3)	
≥ 60	4658 (62.8)	2879 (56.8)	1779 (75.7)	
Smoking, n (%)				< 0.001
Never	4828 (65.1)	3506 (69.2)	1322 (56.3)	
Current	1461 (19.7)	931 (18.4)	530 (22.6)	
Former	1130 (15.2)	633 (12.4)	497 (21.1)	
Drinking, times/week, n (%)				0.085
No	5870 (79.1)	4040 (79.7)	1830 (77.9)	
Yes	1549 (20.9)	1030 (20.3)	519 (22.1)	
Physical activity, n (%)				0.002
No	6117 (82.5)	4133 (81.5)	1984 (84.5)	
Yes	1302 (17.5)	937 (18.5)	365 (15.5)	
BMI, kg/m^2^, *n* (%)				0.842
non-Obesity	6645 (89.6)	4544 (89.6)	2101 (89.4)	
Obesity	774 (10.4)	526 (10.4)	248 (10.6)	
Hypertension, n (%)				< 0.001
No	4913 (66.2)	3429 (67.6)	1484 (63.2)	
Yes	2506 (33.8)	1641 (32.4)	865 (36.8)	
Hyperlipidemia, n (%)				< 0.001
No	7008 (94.5)	4756 (93.8)	2252 (95.9)	
Yes	411 (5.5)	314 (6.2)	97 (4.1)	
Diabetes, n (%)				< 0.001
No	6660 (89.8)	4629 (91.3)	2031 (86.5)	
Yes	759 (10.2)	441 (8.7)	318 (13.5)	
FPG, mg/dL*	89.20 (80.01, 101.99)	88.66 (79.83, 100.37)	90.28 (80.91, 107.04)	< 0.001

AIP index, Atherogenic Index of Plasma; TyG index, Triglyceride-glucose index; TG/HDL-C ratio, Triglyceride-to-high-density lipoprotein cholesterol ratio; METS-IR, Metabolic Score for Insulin Resistance; BMI, body mass index; FPG, Fasting Plasma Glucose. *Data are expressed as median (interquartile range: 25th -75th percentile).

As demonstrated in **Supplementary Data Sheet 1**, the kernel density curves for all four standardized indicators demonstrate unimodal distributions, with a peak near zero. Of particular note are the curves for AIP index and TyG index, which exhibit approximate symmetry and proximity to normality. METS-IR demonstrates a slight right skew, while TG/HDL-C ratio displays a pronounced right skew with a protracted right tail, suggesting the presence of extreme high values. In view of this distributional characteristic, the natural logarithm of the TG/HDL-C ratio was applied prior to its incorporation into subsequent analyses. **Supplementary Data Sheet 1** illustrates the shape of the ln (TG/HDL-C) ratio following natural logarithmic transformation.

### 3.2 Association between alternative indices of IR and CHD

The associations between alternative indices of IR and CHD are presented in **Table 2**. Odds ratios expressed ‘per one-unit increase’ represent the change in CHD risk associated with a one-unit increment in the corresponding index value. In analyses using tertiles of each index, a ‘per tertile increase’ corresponds to an average increment of 0.508 units for AIP index, 1.230 units for TyG index, 13.449 units for METS-IR and 1.169 units for ln(TG/HDL-C) on their original scales. All alternative indices of IR demonstrated a significant positive correlation with the risk of CHD. In the multivariate logistic regression analysis, this association remained significant after adjusting for age, gender, BMI, drinking, smoking, physical activity, hypertension, and hyperlipidemia in model 3. When insulin resistance indices were treated as continuous variables, the AIP index (*OR*: 1.612, 95% *CI*: 1.333, 1.951), TyG index (*OR*: 1.208, 95% *CI*: 1.101, 1.325), METS-IR (*OR*: 1.039, 95% *CI*: 1.030, 1.048), and ln(TG/HDL-C) (*OR*: 1.230, 95% *CI*: 1.133, 1.337) were all found to be significant. Furthermore, higher levels of the surrogate marker for IR, whether expressed as a tertile or per tertile increase, were associated with a greater likelihood of CHD. The robustness of this association was consistent across various adjustment models.

**Table 2:** Logistics regression analysis was conducted to assess the relationship between alternative indexes of insulin resistance and coronary heart disease.

Alternative indexes of insulin resistance	*OR (95%CI)*		
	Model 1	Model 2	Model 3
AIP index			
Per one-unit increase	1.621 (1.355, 1.941)	1.646 (1.365, 1.986)	1.612 (1.333, 1.951)
Tertile			
T1	Ref.	Ref.	Ref.
T2	1.267 (1.060, 1.514)	1.219 (1.014, 1.465)	1.213 (1.008, 1.460)
T3	1.566 (1.299, 1.888)	1.544 (1.272, 1.873)	1.521 (1.250, 1.850)
*P*trend	< 0.001	< 0.001	< 0.001
Per tertile increase	1.278 (1.167, 1.400)	1.288 (1.171, 1.416)	1.274 (1.157, 1.404)
TyG index			
Per one-unit increase	1.126 (1.032, 1.229)	1.192 (1.089, 1.305)	1.208 (1.101, 1.325)
Tertile			
T1	Ref.	Ref.	Ref.
T2	1.076 (0.936, 1.237)	1.133 (0.981, 1.310)	1.140 (0.985, 1.319)
T3	1.198 (0.996, 1.442)	1.338 (1.104, 1.620)	1.374 (1.129, 1.669)
*P*trend	< 0.001	0.003	< 0.001
Per tertile increase	1.158 (1.040, 1.289)	1.242 (1.111, 1.388)	1.261 (1.126, 1.413)
METS-IR			
Per one-unit increase	1.032 (1.024, 1.039)	1.030 (1.023, 1.038)	1.039 (1.030, 1.048)
Tertile			
T1	Ref.	Ref.	Ref.
T2	1.501 (1.323, 1.702)	1.450 (1.273, 1.651)	1.445 (1.268, 1.648)
T3	1.761 (1.478, 2.100)	1.737 (1.448, 2.085)	1.913 (1.541, 2.375)
*P*trend	< 0.001	< 0.001	< 0.001
Per tertile increase	1.525 (1.383, 1.681)	1.497 (1.352, 1.657)	1.672 (1.481, 1.887)
ln (TG/HDL-C)			
Per one-unit increase	1.234 (1.141, 1.334)	1.242 (1.145, 1.347)	1.230 (1.133, 1.337)
Tertile			
T1	Ref.	Ref.	Ref.
T2	1.267 (1.060, 1.514)	1.219 (1.014, 1.465)	1.213 (1.008, 1.460)
T3	1.566 (1.299, 1.888)	1.544 (1.272, 1.873)	1.521 (1.251, 1.850)
*P*trend	< 0.001	< 0.001	< 0.001
Per tertile increase	1.278 (1.167, 1.400)	1.288 (1.171, 1.416)	1.274 (1.157, 1.404)

OR, Odd ratios; CI, confidence interval; T1, First tertile; T2, Second tertile; T3, Third tertile; AIP index, Atherogenic Index of Plasma; TyG index, triglyceride glucose index; METS-IR, metabolic score for Insulin resistance; ln (TG/HDL-C), natural logarithm of the triglyceride to high-density lipoprotein cholesterol ratio.

Model 1: adjusted for none;

Model 2: general demographic characteristics (age, gender) were adjusted;

Model 3: adjusted for BMI, medical history (hypertension, hyperlipidemia) and behavioral risk factors (drinking, smoking, physical activity) on the basis of model 2;

*P* trend: calculated according to tertile order rank.

**Figure 1:**
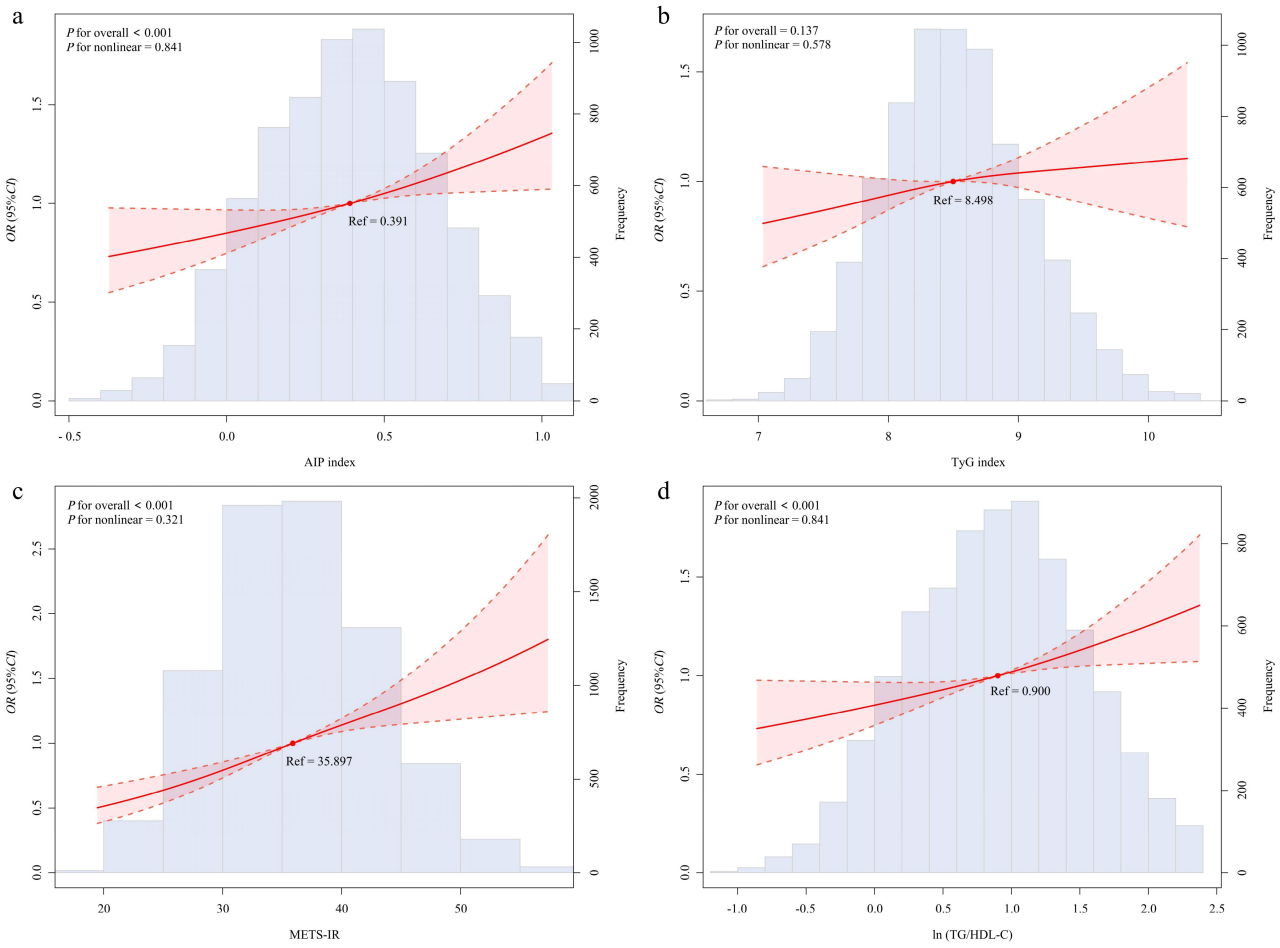
Dose–response relationship of insulin resistance-related indices with the risk of CHD modeled by restricted cubic splines. Models were adjusted for demographic characteristics (age, gender), BMI, medical history (hypertension, hyperlipidemia) and behavioral risk factors (drinking, smoking, physical activity). Solid lines represent odds ratios, dashed lines represent 95% confidence intervals, and histograms show the distribution of insulin resistance-related indices. Restricted cubic splines were used to visualize the shape of the associations, whereas statistical inference is based on the prespecified multivariable logistic regression model 3. *OR*, Odd ratios; *CI*, confidence interval; AIP index, Atherogenic Index of Plasma; TyG index, triglyceride glucose index; METS-IR, metabolic score for Insulin resistance; ln (TG/HDL-C), natural logarithm of the triglyceride to high-density lipoprotein cholesterol ratio. **(a)** AIP index, **(b)** TyG index, **(c)** METS-IR, **(d)** ln (TG/HDL-C).

The exposure-response relationship between surrogate markers of IR and CHD is illustrated in **Figure 1**. A notable shift in surrogate markers of IR was observed at values of 0.391 (AIP index), 8.498 (TyG index), 35.897 (METS-IR), and 0.900 (ln (TG/HDL-C)). There was no evidence of nonlinearity (P-nonlinear>0.05) with four indexes, consistent with an approximately linear, modest increase across the observed range.

### 3.3 Subgroup and interaction analyses

**Figure 2:**
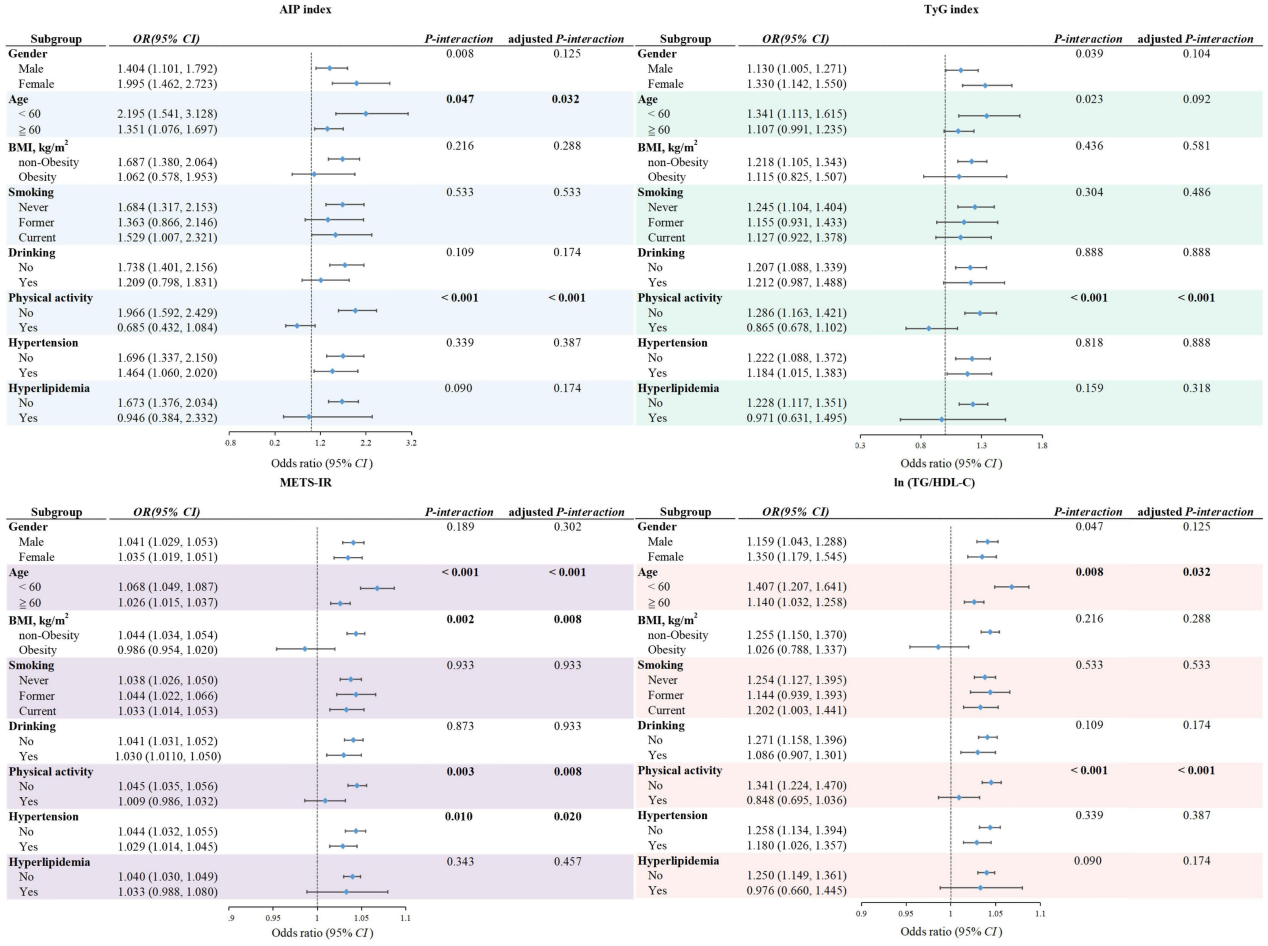
Association of alternative indexes of insulin resistance with coronary heart disease stratified by participant characteristics. Each stratification controlled for all factors (age, gender, BMI, smoking, drinking, physical activity, hypertension and hyperlipidemia) except the stratification factor itself. *P*-interaction was assessed by combining the variables’ cross-product term (alternative indexes of insulin resistance × characteristics) in the same model. *OR*, Odd ratios; 95% *CI*, 95% confidence intervals; AIP index, Atherogenic Index of Plasma; TyG index, triglyceride glucose index; METS-IR, metabolic score for Insulin resistance; ln (TG/HDL-C), natural logarithm of the triglyceride to high-density lipoprotein cholesterol ratio.

Subgroup and interaction analyses stratified by age, gender, BMI, drinking, smoking, physical activity, hypertension, and hyperlipidemia as shown in **Figure 2**. Across most strata, higher levels of each IR surrogate were consistently associated with a higher prevalence of CHD. After applying the FDR procedure to the interaction tests, all physical activity × IR interactions remained statistically significant (adjusted *P*-interaction < 0.05). For age, FDR-adjusted interaction *P*-values remained below 0.05 for the AIP index, METS-IR and ln(TG/HDL-C), with larger effect estimates in participants aged < 60 years, whereas the age × TyG interaction was attenuated and no longer reached conventional statistical significance after FDR correction(adjusted *P*-interaction > 0.05).

### 3.4 Potential pathways and mediation analyses

**Figure 3:**
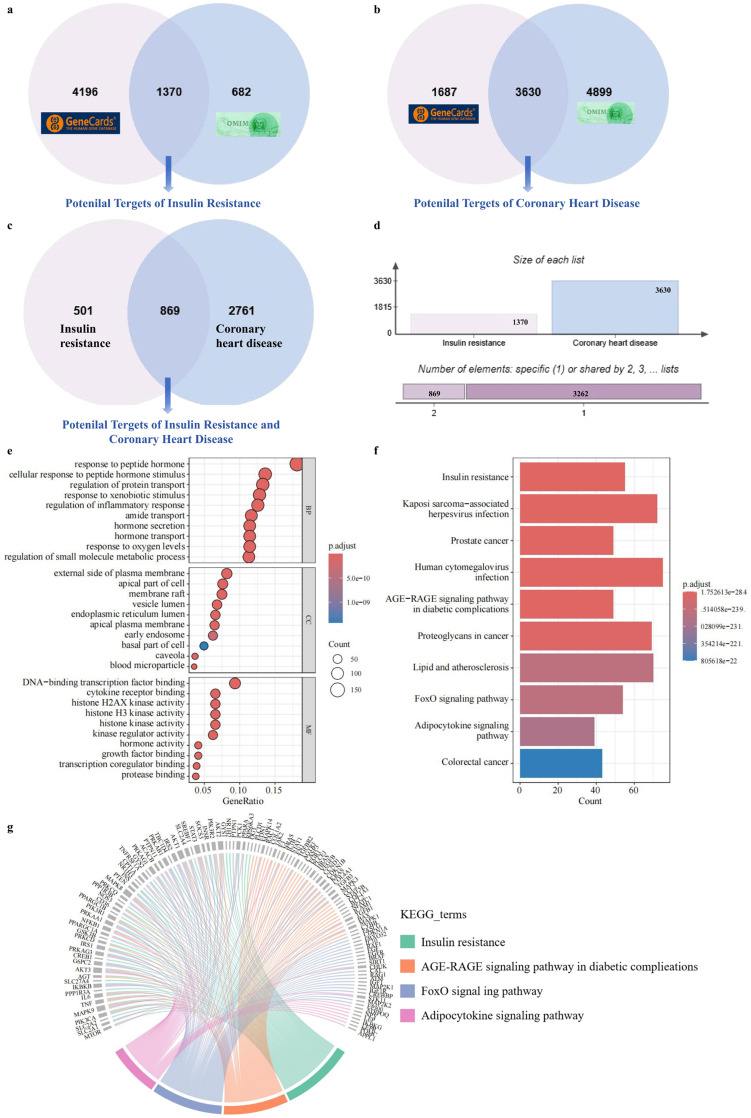
Acquisition of potential pathways linking insulin resistance and coronary heart disease. **(A, B)** Venn diagrams showing potential target genes associated with insulin resistance and coronary heart disease, respectively. **(C, D)** Overlapping targets between the two conditions, with 869 shared genes indicating potential biological connections. **(E, F)** GO and KEGG enrichment analyses of the common targets, revealing key biological processes and signaling pathways. **(G)** Chord diagram visualizing genes involved in KEGG-enriched pathways shared by both conditions.

In order to further investigate the potential mechanisms underlying this association, 1,370 potential targets for IR and 3,630 potential targets for CHD were identified, with 869 genes present in both diseases (**Figures 3A–D**). Enrichment analysis of these common targets revealed significant enrichment in pathways related to hormone response, inflammatory regulation, and membrane transport (**Figure 3E**). The pathway analysis indicated significant involvement of IR, the advanced glycation end-product (AGE)-receptor signaling pathway, lipid metabolism and atherosclerosis, FoxO, and adipokine pathways (**Figure 3F**). The full KEGG and GO enrichment output (including term, gene count, P and FDR) as **Supplementary Data Sheets 3, 4**. String diagrams revealed substantial overlap among IR, AGE-receptor, FoxO, and adipokine signaling pathways, with functional enrichment observed in genes associated with fasting blood glucose across these pathways (**Figure 3G**).

**Figure 4:**
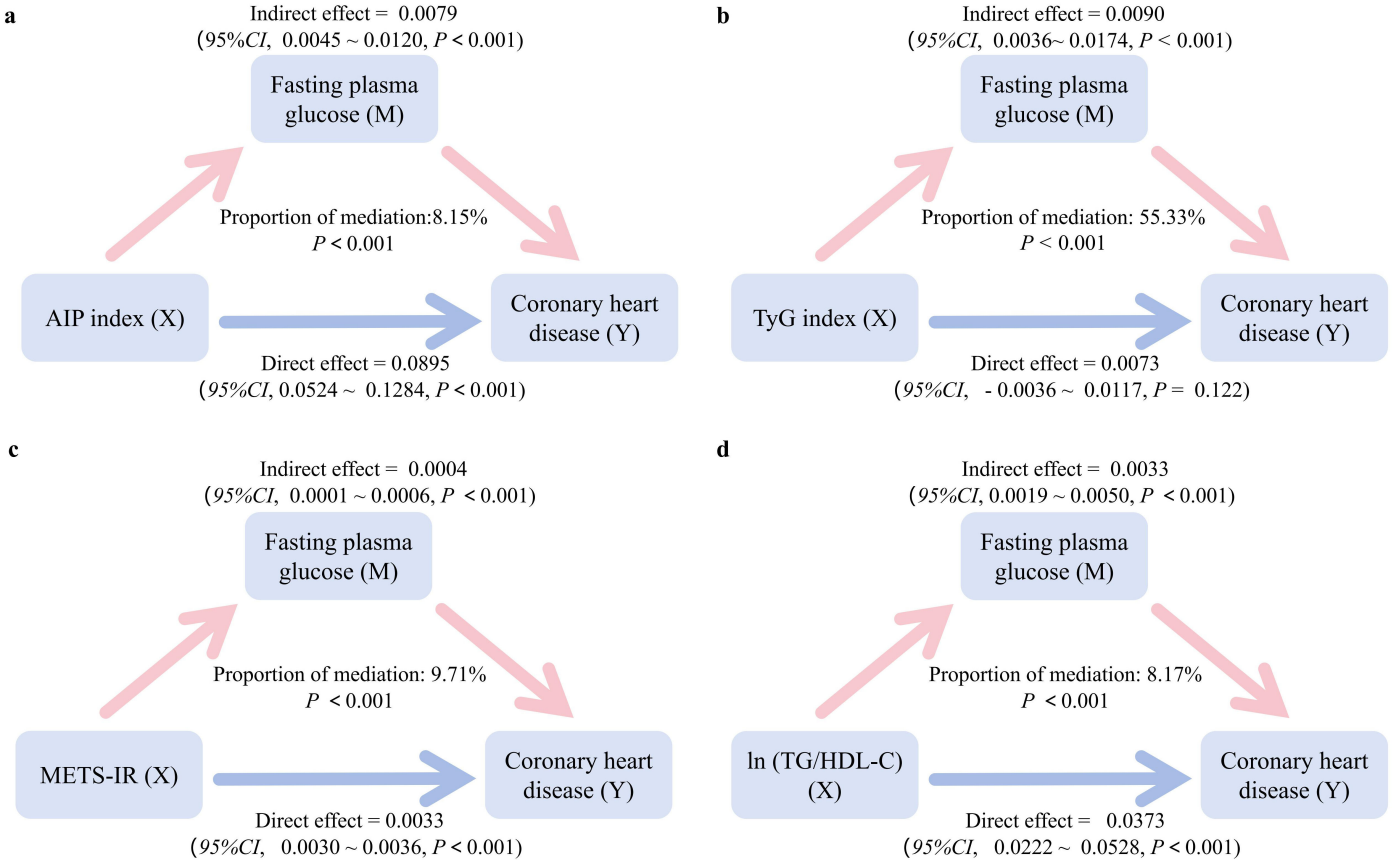
Association between insulin resistance-related indices, fasting plasma glucose as a mediator and coronary heart disease. Mediation Models controlled for age, gender, BMI, smoking, drinking, physical activity, hypertension and hyperlipidemia. The average causal mediation effect (ACME) represents the effects of the insulin resistance-related indices on coronary heart disease prevalence through the mediator. Proportion of mediation was calculated via dividing ACME by TE (total effect). 95% *CI*, 95% confidence intervals; AIP index, Atherogenic Index of Plasma; TyG index, triglyceride glucose index; METS-IR, metabolic score for Insulin resistance; ln (TG/HDL-C), natural logarithm of the triglyceride to high-density lipoprotein cholesterol ratio. **(a)** AIP index, **(b)** TyG index, **(c)** METS-IR, **(d)** ln (TG/HDL-C).

Pathway enrichment analysis indicates FoxO as one of the potential pathways. Previous studies suggest FoxO primarily influences fasting blood glucose by directly inhibiting hepatic gluconeogenesis; suppression of hepatic FoxO reduces fasting blood glucose levels (30, 31). The glycated end-product receptor activator pathway interacts with inflammatory gene networks. Randomized controlled trials indicate that limiting glycated end-products can reduce inflammation, improve glucose metabolism, and lower fasting blood glucose (32, 33). Adipokine signaling enhances insulin sensitivity and glucose uptake, consistent with reduced FPG outcomes in human studies (34, 35). In summary, these pathways collectively influence FPG regulation; consequently, we selected FPG as the mediating indicator for assessing IR’s impact on CHD in subsequent analyses. The mediation analysis in **Figure 4** results indicated that FPG mediated 8.15%, 55.33%, 9.71%, and 8.17% of the association effects between AIP index, TyG index, METS-IR, ln(TG/HDL-C) and CHD, respectively. Absolute ACME, ADE and total effect values with CIs for each IR index were shown in **Supplementary Table 3** of **Supplementary Data Sheet 1**.

### 3.5 Sensitivity analyses

In order to assess the robustness of the model, several sensitivity analyses were conducted. Re-analysis of the pre-imputation dataset revealed that the associations between the four indicators and CHD remained stable after full covariate adjustment (**Supplementary Data Sheet 1**). Further adjusting the source of participants on the basis of the fully adjusted Model 3, the associations between each IR surrogate and prevalent CHD were unchanged, and all associations remained statistically significant (*P* < 0.05) (**Supplementary Data Sheet 1**). In analyses stratified by recruitment source, all four IR indices showed positive associations with CHD among cardiology inpatients, whereas effect estimates in the health-examination subgroup were close to null with wide confidence intervals that all included 1. Interaction *P*-values were <0.05 for AIP index and ln(TG/HDL-C), but not for TyG index or METS-IR, indicating that any heterogeneity by recruitment source should be interpreted with caution given the limited power in the health-examination subgroup. (**Supplementary Data Sheet 1**). Subsequently, we employed the inverse probability weighting method and managed extreme weight values by truncating the distribution at the 1st and 99th percentiles. The results indicated that there were no substantial alterations in any associations (**Supplementary Data Sheet 1**). Following the exclusion of individuals diagnosed with diabetes, the results for all indicators remained stable, with the exception of TyG index, as illustrated in the **Supplementary Data Sheet 1**. In order to assess the robustness of the mediation analysis findings, outer-layer random subsampling was performed (500 iterations, with 80% of the sample being drawn each time) under the probit link function. The mediation proportions were consistent with the main findings (**Supplementary Data Sheet 1**).

## 4 Discussion

In this large cross-sectional study, we observed that four non-insulin-based surrogate indices of IR: AIP index, TyG index, METS-IR and ln(TG/HDL) and were each significantly associated with the prevalence of CHD. We also observed that physical inactivity was the predominant effect modifier (interaction *P*-value < 0.05), with a possible additional influence of younger age, so that sedentary adults <60 years with elevated IR surrogates appeared particularly vulnerable. Mechanistically, bioinformatic enrichment analyses highlighted three core pathways, including AGE–RAGE signaling in diabetic complications, FoxO signaling, and adipocytokine signaling, converge on glycemic regulation, implicating disordered glucose metabolism as a plausible mechanism underlying these associations. Consistent with this, when FPG was evaluated as a mediator, it partially accounted for the observed relationships.

Our findings are broadly consistent with previous studies linking TyG, METS-IR, AIP and TG/HDL-C to cardiovascular events, CHD severity and cardiovascular mortality in diverse populations (12–16). Crucially, because these metrics are computed from routine fasting glucose and lipid panels, they do not require insulin assays, rendering them inexpensive, scalable, and readily implementable in both clinical and population settings, an advantage that highlights their practical value as simple alternatives to insulin-based measures in routine cardiovascular risk assessment. These results align with prior evidence showing that indices derived from standard biochemistry capture atherosclerosis-relevant metabolic disturbances: for example, higher TyG has predicted ischemic heart disease in both prospective and cross-sectional cohorts: Korean adults without diabetes in the highest TyG quartile had more than twice the risk of ischemic heart disease compared with those in the lowest quartile (12), and Chinese patients undergoing coronary angiography with higher TyG had more than double the odds of multivessel disease (13), these associations have been replicated across 22 countries in PURE (14). Likewise, the TG/HDL-C ratio, which is closely related to AIP, has been linked to incident ischemic heart disease, with approximately a twofold higher risk in the highest versus the lowest quartile (15), AIP has been associated with rapid coronary atherosclerosis progression beyond traditional risk factors (16), and METS-IR has shown robust predictive capacity in longitudinal studies. Taken together, our results support the use of any of these indices as convenient, low-cost proxies for IR in both clinical and epidemiological cardiovascular risk assessment.

In the subgroup analysis, physical active modified the IR–CHD association: relative odds per unit increase in any non–insulin-based IR surrogate were higher in physically inactive than in physically active participants. Higher leisure time, occupational and commuting activities were prospectively associated with lower CVD risk and improved insulin sensitivity (36, 37), which is similar to previous findings. While age-related differences were more modest, most IR surrogates still showed higher effect estimates in adults < 60 than in those ≥60 years. Based on previous research, TyG predicting mortality chiefly in < 65-year groups (38), a stronger inverse link between insulin sensitivity and carotid atherosclerosis progression in younger/middle-aged men (39); and METS-IR predicting mortality predominantly in non-elderly adults (11). Mechanistically, longer lifetime exposure to dysglycemia and early adverse metabolic profiles may amplify risk in younger individuals (11, 40), whereas multimorbidity in older adults may attenuate IR’s marginal impact. We therefore regard age as a possible effect modifier, but consider these age-related findings, particularly for TyG, to be suggestive and hypothesis-generating rather than definitive. In summary, sedentary, non-elderly adults with elevated IR surrogates appear particularly vulnerable; integrating TyG, METS-IR, AIP, or TG/HDL-C into routine assessments may help flag candidates for targeted lifestyle interventions.

The biological mechanisms linking IR to CHD are complex and multifactorial. IR promotes a constellation of pro-atherogenic perturbations, including dysglycemia, atherogenic dyslipidemia, hypertension, and low-grade inflammation, which act in concert to accelerate atherogenesis (41, 42). A central question is how much of the IR–CHD relationship is transmitted through hyperglycemia. In IR, impaired insulin signaling fails to suppress hepatic gluconeogenesis and reduces muscle glucose uptake, resulting in persistently elevated fasting glucose levels (43). This chronic hyperglycemia initiates vascular injury by increasing reactive oxygen species and promoting inflammation in endothelial cells and macrophages (44)and drives advanced glycation end-products (AGEs) that bind to RAGE on vascular cells, amplifying inflammatory cascades (45), consistent with clinical links between poor long-term glycemic control and impaired endothelium-dependent vasodilation (46). The surrogate indices used in our study, capture these underlying processes: AIP reflects lipid-driven atherogenesis, while TyG and METS-IR incorporate fasting glucose and are closely related to visceral adiposity and systemic inflammation. Genomic studies further implicate the AGE–RAGE, FoxO, adipocytokine, and lipid metabolism pathways in linking IR to atherosclerosis (47). In IR, sustained FoxO activity promotes gluconeogenesis and oxidative stress in vascular cells (48, 49), while impaired adipocytokine signaling favors inflammation and endothelial injury (49, 50). Together, these lines of evidence motivated our mediation analysis using FPG (**Figure 4**), which supports a partial glycemic pathway underscoring that even modest FPG elevations in at-risk individuals may warrant early intervention to mitigate long-term cardiovascular risk.

Several limitations of our study should be acknowledged. First, the cross-sectional, hospital-based design in an ethnically homogeneous (East Asian, predominantly Chinese) sample of mainly middle-aged and older adults limits both causal inference and external generalizability. Although we adjusted for multiple known risk factors and comorbidities, residual confounding and selection bias cannot be ruled out, and our findings may not be directly generalizable to younger individuals, other ethnic groups or non-hospital populations in China. Prospective, multi-center studies in more diverse populations are needed to confirm whether elevated IR surrogates prospectively predict incident CHD events and to establish population-specific cut-off values. Second we focused on surrogate markers and did not have direct measurements of insulin (hence we could not calculate HOMA-IR or quantify IR in absolute terms). However, prior studies indicate that indices like TyG and METS-IR correlate well with directly measured insulin sensitivity and even outperform insulin-based indices (e.g. HOMA-IR) in predicting certain outcomes (7). Third, we did not have information on current use, dose, or duration of cardiometabolic medications (e.g. statins, antihypertensive drugs, antiplatelet agents, or glucose-lowering therapies), and therefore could not adjust directly for these treatments. Our fully adjusted models included medical history of hypertension and hyperlipidemia, and a sensitivity analysis excluding participants with diabetes yielded effect estimates broadly consistent with the main results, but residual confounding by medication use cannot be ruled out. Given that such therapies are more likely to be prescribed to individuals at higher baseline cardiovascular risk (51–53), this limitation probably biased our estimates toward underestimating the true strength of the associations between insulin-resistance indices and CHD (54).

Despite these limitations, our study has several strengths, including the large sample size, side-by-side evaluation of multiple non–insulin-based IR surrogates, and the integration of bioinformatic pathway and mediation analyses, which together provide a coherent picture linking insulin resistance, glycemic pathways and prevalent CHD in this regional hospital-based studies. Looking forward, our findings suggest key research and clinical directions. Longitudinal cohorts and intervention trials are needed to determine whether improving IR reduces CHD risk. Another avenue is incorporating metabolic risk markers into clinical assessment. Indices like TyG and TG/HDL-C, easily calculated from routine labs, could be integrated into cardiovascular risk screening.

## 5 Conclusion

Our findings demonstrate that IR surrogates, including the AIP index, TyG index, METS-IR, and ln(TG/HDL-C) ratio, are significantly associated with an increased prevalence of CHD. The strength of these associations appears to be influenced by physical activity, and possibly by age, with more pronounced effects in younger and physically inactive individuals. Bioinformatic enrichment analyses further converged on glycemic axes, highlighting AGE–RAGE signaling in diabetic complications, FoxO signaling, and adipocytokine signaling, thereby providing mechanistic context for the epidemiologic patterns. Furthermore, mediation analysis indicates that FPG partially mediates the relationship between IR surrogates and CHD, accounting for 8.15% to 55.33% of the observed effect. Collectively, these findings support the potential clinical utility of simple, insulin-independent indices for identifying hospital-based populations with a higher prevalence of CHD and highlight glycemic pathways and physical inactivity as important correlates that warrant further investigation in longitudinal studies.

## Data Availability

The original contributions presented in the study are included in the article/**Supplementary Materials**. Further inquiries can be directed to the corresponding author.
