# Supplementary material for: Insulin resistance and its association with coronary heart disease: insights from bioinformatics analysis
Source: Front Endocrinol (Lausanne). 2025 Dec 12;16:1730801. doi: 10.3389/fendo.2025.1730801 (PMC12740753; doi:10.3389/fendo.2025.1730801)
Supplement: Supplementary file 1 [file DataSheet1.docx]

**Supplement information**

**Insulin resistance and coronary heart disease in southeastern China: insights from bioinformatics analysis**

**Xinyue Lu ^1†^, Minghuan Liu ^1†^, Yang Song ^1†^, Shiying Lin ^1^, Xiaowan Zhou^1^, Hang Zeng^1^, Aina Li ^2^, Huangyuan Li** **^3^, Xiaoxu Xie ^1*^, Shaowei Lin ^1*^, Siying Wu^1*^**

**1 Department of Epidemiology and Health Statistics, The School of Public Health, Fujian Medical University, Fuzhou, 350122, China**

**2 Department of Cardiology, The First Affiliated Hospital of Fujian Medical University, Fuzhou 350001, China.**

**3 Department of Preventive Medicine, The School of Public Health, Fujian Medical University, Fuzhou, China**

***Corresponding authors at. Department of Epidemiology and Health Statistics, School of Public Health, Fujian Medical University, Minhou County, Fuzhou, 350122, China (**[fmuwsy@163.com](mailto:fmuwsy@163.com)**, linsw@fjmu.edu.cn, xxx@fjmu.edu.cn).**

**^†^ These authors contributed equally to this work.**

Number of Pages: 23

Number of Supplementary Methods: 2

Number of Tables: 8

Number of Figures: 5

**Contents**

**Supplementary Method.** Details about MICE interpolation

**Supplementary Method.** Details about mediation analyses

**Supplementary Table 1.** Coding of all exposures and covariates in the analysis

**Supplementary Table 2.** Descriptive statistics of the study population with difference source.

**Supplementary Table 3.** Results from mediation analyses, shown as the ACME, ADE and total effects.

**Supplementary Table 4.** Logistics regression analysis was conducted to assess the relationship between alternative indexes of insulin resistance and coronary heart disease without imputing the missing data of covariates.

**Supplementary Table 5.** Adjusted odds ratios (95% CIs) for coronary heart disease according to alternative insulin resistance indicators, with further adjustment for the source of study participants.

**Supplementary Table 6.** Associations between alternative indexes of insulin resistance and coronary heart disease in analyses stratified by participant sources.

**Supplementary Table 7.** Odd ratios (95%*CI*s) of coronary heart disease associated with alternative indexes of insulin resistance using ipws (truncating at 1st and 99th percentile of weights distribution)

**Supplementary Table 8.** Logistics regression analysis was conducted to assess the relationship between alternative indexes of insulin resistance and coronary heart disease excluding participants with diabetes.

**Supplementary Figure 1.** Flowchart of participants recruitment and selection.

**Supplementary Figure 2.** Trace plots for key covariates in the multiple imputation procedure.

**Supplementary Figure 3.** Distribution of four standardized alternative indexes of insulin resistance using kernel density estimation**.**

**Supplementary Figure 4.** Distribution of standardized ln (TG/HDL-C) using kernel density estimation.

**Supplementary Figure 5**. Probit-based sensitivity analysis demonstrating the robustness of the primary logit results.

**Supplementary Method. Details about MICE interpolation**

Missing values in covariates were handled using multivariate imputation by chained equations (MICE), implemented in the mice package in R (version 3.18.0). We generated five imputed datasets (m = 5) using five iterations of the chained-equations algorithm (maxit = 5), which is generally sufficient for convergence in moderately sized epidemiological datasets.

The imputation model included smoking, drinking, metabolic equivalent of task, systolic and diastolic blood pressure, and total cholesterol. For all continuous and categorical covariates included in the imputation model we used predictive mean matching (method = "pmm"), in order to preserve the observed data distribution and avoid implausible imputed values. For reproducibility, we set a global seed before imputation (set.seed(12345)) and additionally specified a seed within the mice() call (seed = 123), ensuring that the same imputed datasets can be regenerated.

Convergence of the MICE algorithm was assessed using the standard trace plots provided by mice (**Supplementary Figure 1**). For covariates (smoking, drinking, metabolic equivalent of task, systolic and diastolic blood pressure, and total cholesterol), we visually inspected chain-specific means and standard deviations across the five iterations for each of the five imputation chains. The traces showed good mixing between chains and random fluctuation around stable values, without systematic upward or downward trends, indicating satisfactory convergence of the imputation procedure.

**Supplementary Method.** **Details about mediation analyses**

To investigate whether fasting plasma glucose (FPG) mediates the associations between insulin resistance (IR) surrogates and CHD, we conducted causal mediation analyses using the R package mediation. For each IR index X, we specified a linear regression model for the mediator and a logistic regression model for the outcome as follows:

- Mediator model (linear regression, Gaussian family):

$$\text{ }\text{FPG}\text{ }\text{=}{\text{ }\text{β}}_{\text{0}}\text{ }\text{+}\text{ }\text{β}_{1}\text{X}\text{ }\text{+}\text{ }\beta_{2}\text{ }\text{Age}\text{ +}\text{ }\text{β}_{3}\text{ }\text{G}\text{ender}\text{ }\text{+}\text{ }\text{β}_{4}\text{ }\text{BMI}\text{ }\text{+}\text{ }\text{β}_{5}\text{ }\text{Smoking}\text{ }\text{+}\text{ }\text{β}_{6}\text{ }\text{Drinking}\text{ }$$

$$\text{ }\text{+}\text{ }\text{β}_{7}\text{ }\text{Physical activity}\text{+}\text{ }\text{β}_{8}\text{ }\text{Hypertension}\text{ }\text{+}\text{ }\text{β}_{9}\text{ }\text{Hyperlipidemia}\text{ }\text{+}\text{ }\text{ε}\text{ }$$

- Outcome model (logistic regression, binomial family):

$$\text{logit}\text{\{}\text{P}\text{(CHD}\text{ }\text{=}\text{ }\text{1)}\text{\}}\text{ }\text{=}\text{ }\text{α}_{0}\text{ }\text{+}\text{ }\text{α}_{1}\text{ }\text{X}\text{ }\text{+}\text{ }\text{α}_{2}\text{ }\text{FPG}\text{ }\text{+}\text{ }\text{α}_{3}\text{ }\text{Age}\text{ }\text{+}\text{ }\text{α}_{4}\text{ G}\text{ender}\text{ }\text{+}\text{ }\text{α}_{5}\text{ }\text{BMI}\text{ }\text{+}\text{ }\text{α}_{6}\text{ }\text{Smoking}\text{ }$$

$$\text{ + }\text{α}_{7}\text{ Drinking + }\text{α}_{8}\text{ Physical activity + }\text{α}_{9}\text{ Hypertension + }\text{α}_{10}\text{Hyperlipidemia.​ }$$

Age, gender, BMI, smoking, drinking, physical activity, hypertension and hyperlipidemia were coded in the same way as in the fully adjusted logistic regression models. FPG was treated as a continuous mediator and each IR index was treated as a continuous exposure. For each IR index, we fitted the mediator and outcome models and then applied the mediate() function to estimate the **average causal mediation effect (ACME, indirect effect via FPG)**, the **average direct effect (ADE, effect not through FPG)**, the **total effect** (ACME + ADE), and the **proportion mediated** (ACME / total effect × 100%). We assumed no exposure–mediator interaction in the outcome model. Confidence intervals and *P* values were obtained using **non-parametric bootstrap with 1,000 resamples** (boot = TRUE, sims = 1000). Because the outcome model used a logit link, ACME, ADE and total effects are reported on the **risk-difference scale**, that is, as **absolute differences in the predicted probability of CHD associated with a one-unit increase in each IR index**.

Supplementary Table 1. Coding of all exposures and covariates in the analysis

| Variable name | Coding |
| --- | --- |
| Tertiles of IR indices | 0 = lowest tertile; 1 = middle tertile;  2 = highest tertile |
| Gender | 0 = female; 1 = male |
| Age | 0 = < 60; 1 = ≧ 60 |
| Smoking | 0 = never smoking;  1 = current smoking;  2 = former smoking |
| drinking | 0 = No; 1 = Yes |
| Physical activity | 0 = No;1 = Yes |
| BMI | 0 = < 28 ; 1 = ≧ 28 |
| Hypertension | 0 = No; 1 = Yes |
| Hyperlipidemia | 0 = No; 1 = Yes |

Supplementary Table 2. Descriptive statistics of the study population with difference source.

| **Characteristics** | **Cardiology inpatient department**  **(*n* = 4,788)** | **Health examination centre**  **(*n* = 2,631)** | ***P* value** |
| --- | --- | --- | --- |
| **CHD, *n* (%)** |  |  | < 0.001 |
| No | 2504 (52.3) | 2566 (97.5) |  |
| Yes | 2284 (47.7) | 65 (2.5) |  |
| **AIP index*** | 0.40 (0.20, 0.59) | 0.38 (0.18, 0.56) | 0.003 |
| **TyG index*** | 8.47 (8.09, 8.85) | 8.54 (8.21, 8.95) | < 0.001 |
| **TG/HDL-C ratio*** | 2.51 (1.58, 3.90) | 2.37 (1.51, 3.62) | 0.003 |
| **METS_IR*** | 36.44 (31.84, 41.27) | 34.99 (30.74, 39.72) | < 0.001 |
| **Gender, *n* (%)** |  |  | < 0.001 |
| Male | 2812 (58.7) | 1260 (47.9) |  |
| Female | 1976 (41.3) | 1371 (52.1) |  |
| **Age, years , *n* (%)** |  |  | < 0.001 |
| < 60 | 1610 (33.6) | 1150 (43.7) |  |
| ≥ 60 | 3178 (66.4) | 1481 (56.3) |  |
| **BMI, kg/m^2^, *n* (%)** |  |  | 0.178 |
| non-Obesity | 4271 (89.2) | 2374 (90.2) |  |
| Obesity | 517 (10.8) | 257 (9.77) |  |
| **Smoking, *n* (%)** |  |  | < 0.001 |
| Never | 2978 (62.2) | 1849 (70.3) |  |
| Current | 990 (20.7) | 467 (17.7) |  |
| Former | 820 (17.1) | 315 (12.0) |  |
| **Drinking, times/week, *n* (%)** | |  | 0.131 |
| No | 3771 (78.8) | 2112 (80.3) |  |
| Yes | 1017 (21.2) | 519 (19.7) |  |
| **Physical activity, *n* (%)** | |  | < 0.001 |
| No | 4060 (84.8) | 2055 (78.1) |  |
| Yes | 728 (15.2) | 576 (21.9) |  |
| **Hypertension, *n* (%)** | |  | 0.33 |
| No | 3148 (65.7) | 1760 (66.9) |  |
| Yes | 1640 (34.3) | 871 (33.1) |  |
| **Hyperlipidemia, *n* (%)** | |  | < 0.001 |
| No | 4594 (95.9) | 2414 (91.8) |  |
| Yes | 194 (4.1) | 217 (8.2) |  |
| **Diabetes, *n* (%)** |  |  | 0.042 |
| No | 4324 (90.3) | 2336 (88.8) |  |
| Yes | 464 (9.7) | 295 (11.2) |  |
| **FPG, mg/dL*** | 86.86 (77.85, 100.19) | 92.98 (84.33, 104.70) | < 0.001 |

Abbreviations: CHD: Coronary heart disease; AIP index: Atherogenic Index of Plasma; TyG index: Triglyceride-glucose index; TG/HDL-C ratio: Triglyceride-to-high-density lipoprotein cholesterol ratio; METS-IR: Metabolic Score for Insulin Resistance; BMI, body mass index; FPG: Fasting Plasma Glucose. *Data are expressed as median (interquartile range: 25th -75th percentile).

Supplementary Table 3. Results from mediation analyses, shown as the ACME, ADE and total effects.

| **Alternative indexes  of insulin resistance** | **Effect type** | **Estimate** | **95% CI lower** | **95% CI upper** |
| --- | --- | --- | --- | --- |
| **AIP index** | ACME | 0.0079 | 0.0045 | 0.0120 |
|  | ADE | 0.0895 | 0.0524 | 0.1284 |
|  | total effects | 0.0974 | 0.0596 | 0.1368 |
| **TyG index** | ACME | 0.0090 | 0.0036 | 0.0174 |
|  | ADE | 0.0073 | −0.0036 | 0.0117 |
|  | total effects | 0.0164 | 0.0125 | 0.0169 |
| **METS-IR** | ACME | 0.0004 | 0.0001 | 0.0006 |
|  | ADE | 0.0033 | 0.0030 | 0.0036 |
|  | total effects | 0.0037 | 0.0035 | 0.0038 |
| **ln(TG/HDL-C)** | ACME | 0.0033 | 0.0019 | 0.0050 |
|  | ADE | 0.0373 | 0.0222 | 0.0528 |
|  | total effects | 0.0406 | 0.0252 | 0.0562 |

Abbreviation: ACME, average causal mediation effect (indirect effect via fasting plasma glucose); ADE, average direct effect (effect not through fasting plasma glucose); Total effect = ACME + ADE. All effects are expressed as absolute risk differences in the probability of CHD per one-unit increase in each insulin resistance surrogate. Estimates and 95% confidence intervals are based on causal mediation analyses using a linear regression model for fasting plasma glucose and a logistic regression model for CHD, with 1,000 non-parametric bootstrap simulations. All models were adjusted for age, gender, BMI , smoking, drinking, physical activity, hypertension and hyperlipidaemia.

Supplementary Table 4. Logistics regression analysis was conducted to assess the relationship between alternative indexes of insulin resistance and coronary heart disease without imputing the missing data of covariates.

| Alternative indexes  of insulin resistance | *OR (95%CI)* | | |
| --- | --- | --- | --- |
|  | Model 1 | Model 2 | Model 3 |
| **AIP index** |  |  |  |
| Per one unit increase | 1.621(1.355, 1.941) | 1.644(1.363, 1.983) | 1.637(1.344, 1.994) |
| Tertile |  |  |  |
| T1 | Ref. | Ref. | Ref. |
| T2 | 1.267 (1.060, 1.514) | 1.224 (1.019, 1.472) | 1.236 (1.021, 1.497) |
| T3 | 1.566 (1.299, 1.888) | 1.546 (1.274, 1.876) | 1.567 (1.280, 1.918) |
| *P*_trend_ | < 0.001 | < 0.001 | < 0.001 |
| Per tertile increase | 1.278 (1.162, 1.405) | 1.287 (1.168, 1.417) | 1.284 (1.158, 1.423) |
| **TyG index** |  |  |  |
| Per one unit increase | 1.126(1.032, 1.229) | 1.193(1.090, 1.306) | 1.195(1.087, 1.315) |
| Tertile |  |  |  |
| T1 | Ref. | Ref. | Ref. |
| T2 | 1.076 (0.936, 1.237) | 1.136 (0.983, 1.313) | 1.132 (0.975, 1.315) |
| T3 | 1.198 (0.996, 1.442) | 1.338 (1.104, 1.621) | 1.369 (1.120, 1.673) |
| *P*_trend_ | 0.058 | 0.003 | 0.002 |
| Per tertile increase | 1.158 (1.040, 1.289) | 1.243 (1.109, 1.392) | 1.246 (1.106, 1.403) |
| **METS-IR** |  |  |  |
| Per one unit increase | 1.032(1.024, 1.039) | 1.030(1.023, 1.038) | 1.039(1.029, 1.049) |
| Tertile |  |  |  |
| T1 | Ref. | Ref. | Ref. |
| T2 | 1.501 (1.323, 1.702) | 1.449 (1.272, 1.650) | 1.436 (1.256, 1.643) |
| T3 | 1.761 (1.478, 2.100) | 1.731 (1.443, 2.078) | 1.864 (1.493, 2.328) |
| *P*_trend_ | < 0.001 | < 0.001 | < 0.001 |
| Per tertile increase | 1.524 (1.381, 1.681) | 1.494 (1.350, 1.654) | 1.670 (1.477, 1.887) |
| **ln (TG/HDL-C)** |  |  |  |
| Per one unit increase | 1.234(1.141, 1.334) | 1.241(1.144, 1.346) | 1.239(1.137, 1.349) |
| Tertile |  |  |  |
| T1 | Ref. | Ref. | Ref. |
| T2 | 1.267 (1.060, 1.514) | 1.224 (1.019, 1.472) | 1.236 (1.021, 1.497) |
| T3 | 1.566 (1.299, 1.888) | 1.546 (1.274, 1.876) | 1.567 (1.280, 1.918) |
| *P*_trend_ | < 0.001 | < 0.001 | < 0.001 |
| Per tertile increase | 1.278 (1.162, 1.405) | 1.287 (1.168, 1.417) | 1.284 (1.158, 1.423) |

Abbreviations: *OR*, Odd ratios; *CI*, confidence interval; T1, First tertile; T2, Second tertile; T3, Third tertile; AIP, Atherogenic Index of Plasma; TyG index, triglyceride glucose index; METS-IR, metabolic score for Insulin resistance; ln (TG/HDL-C), natural logarithm of the triglyceride to high-density lipoprotein cholesterol ratio.

Model 1: adjusted for none;

Model 2: general demographic characteristics (age, gender) were adjusted;

Model 3: adjusted for BMI, medical history (hypertension, hyperlipidemia) and behavioural risk factors (drinking, smoking, physical activity) on the basis of model 2;

*P* trend: calculated according to tertile order rank.

Supplementary Table 5. Adjusted odds ratios (95% CIs) for coronary heart disease according to alternative insulin resistance indicators, with further adjustment for the source of study participants.

| **Alternative indexes  of insulin resistance** | ***OR* (95% *CI*)** | ***P* value** |
| --- | --- | --- |
| **AIP index** |  |  |
| Per one-unit increase | 1.693(1.365,2.099) | < 0.001 |
| Tertile |  |  |
| T1 | Reference |  |
| T2 | 1.322 (1.076, 1.624) | 0.007 |
| T3 | 1.638 (1.316, 2.038) | < 0.001 |
| *P* _trend_ | < 0.001 |  |
| Per tertile increase | 1.306(1.171,1.457) | < 0.001 |
| **TyG index** |  |  |
| Per one-unit increase | 1.499(1.35,1.664) | < 0.001 |
| Tertile |  |  |
| T1 | Reference |  |
| T2 | 1.461 (1.246, 1.712) | < 0.001 |
| T3 | 2.188 (1.751, 2.733) | < 0.001 |
| *P* _trend_ | < 0.001 |  |
| Per tertile increase | 1.646(1.447,1.871) | < 0.001 |
| **METS-IR** |  |  |
| Per one-unit increase | 1.033(1.022,1.043) | < 0.001 |
| Tertile |  |  |
| T1 | Reference |  |
| T2 | 1.416 (1.223, 1.641) | < 0.001 |
| T3 | 1.644 (1.293, 2.090) | < 0.001 |
| *P* _trend_ | < 0.001 |  |
| Per tertile increase | 1.541(1.345,1.765) | < 0.001 |
| **ln (TG/HDL-C)** |  |  |
| Per one-unit increase | 1.257(1.145,1.38) | < 0.001 |
| Tertile |  |  |
| T1 | Reference |  |
| T2 | 1.322 (1.076, 1.624) | 0.008 |
| T3 | 1.638 (1.316, 2.038) | < 0.001 |
| *P* _trend_ | < 0.001 |  |
| Per tertile increase | 1.306(1.171,1.457) | < 0.001 |

Abbreviations: *OR*, Odd ratios; *CI*, confidence interval; T1, First tertile; T2, Second tertile; T3, Third tertile; AIP index, Atherogenic Index of Plasma; TyG index, triglyceride glucose index; METS-IR, metabolic score for Insulin resistance; ln (TG/HDL-C), natural logarithm of the triglyceride to high-density lipoprotein cholesterol ratio. Models were adjusted for demographic characteristics (age, gender), BMI, medical history (hypertension, hyperlipidemia), behavioural risk factors (drinking, smoking, physical activity) and participant source.

*P* trend: calculated according to tertile order rank.

Supplementary Table 6. Associations between alternative indexes of insulin resistance and coronary heart disease in analyses stratified by participant sources.

| **Alternative indexes**  **of insulin resistance** | **source of participants** | | | | |
| --- | --- | --- | --- | --- | --- |
|  | **Cardiology**  **inpatient department** |  | **Health**  **examination centre** |  | ***P* interaction** |
|  | ***OR (95%CI)*** |  | ***OR (95%CI)*** |  |  |
| AIP index | 1.785 (1.430,2.227) |  | 0.680 (0.264,1.752) |  | 0.025 |
| TyG index | 1.529 (1.373,1.702) |  | 1.058 (0.659,1.698) |  | 0.153 |
| METS_IR | 1.034 (1.023,1.045) |  | 1.003 (0.959,1.049) |  | 0.300 |
| ln (TG/HDL-C) | 1.286 (1.168,1.416) |  | 0.846 (0.561,1.276) |  | 0.025 |

Each stratification controlled for all factors (age, gender, BMI, smoking, drinking, physical activity, hypertension and hyperlipidemia). *P*-interaction was assessed by combining the variables’ cross-product term (alternative indexes of insulin resistance × participant source) in the same model. Abbreviations: OR, Odd ratios; 95 % CI, 95 % confidence intervals; AIP index, Atherogenic Index of Plasma; TyG index, triglyceride glucose index; METS-IR, metabolic score for Insulin resistance; ln (TG/HDL-C), natural logarithm of the triglyceride to high-density lipoprotein cholesterol ratio.

Supplementary Table 7. Odd ratios (95%*CI*s) of coronary heart disease associated with alternative indexes of insulin resistance using ipws (truncating at 1st and 99th percentile of weights distribution)

| **Alternative indexes  of insulin resistance** | ***OR* (95% *CI*)** | ***P* value** |
| --- | --- | --- |
| **AIP index** |  |  |
| Per one-unit increase | 1.564 (1.269, 1.929) | < 0.001 |
| Tertile |  |  |
| T1 | Reference |  |
| T2 | 1.209 (1.005, 1.453) | 0.044 |
| T3 | 1.474 (1.214, 1.789) | < 0.001 |
| *P* _trend_ | < 0.001 |  |
| Per tertile increase | 1.236 (1.122, 1.361) | < 0.001 |
| **TyG index** |  |  |
| Per one-unit increase | 1.221 (1.091, 1.366) | < 0.001 |
| Tertile |  |  |
| T1 | Reference |  |
| T2 | 1.093 (0.946, 1.262) | 0.223 |
| T3 | 1.299 (1.070, 1.577) | 0.008 |
| *P* _trend_ | 0.008 |  |
| Per tertile increase | 1.211 (1.081, 1.356) | < 0.001 |
| **METS-IR** |  |  |
| Per one-unit increase | 1.035 (1.024, 1.046) | < 0.001 |
| Tertile |  |  |
| T1 | Reference |  |
| T2 | 1.365 (1.188, 1.569) | < 0.001 |
| T3 | 1.976 (1.572, 2.484) | < 0.001 |
| *P* _trend_ | < 0.001 |  |
| Per tertile increase | 1.672 (1.491, 1.875) | < 0.001 |
| **ln (TG/HDL-C)** |  |  |
| Per one-unit increase | 1.202 (1.106, 1.306) | < 0.001 |
| Tertile |  |  |
| T1 | Reference |  |
| T2 | 1.209 (1.005, 1.454) | 0.044 |
| T3 | 1.474 (1.214, 1.788) | < 0.001 |
| *P* _trend_ | < 0.001 |  |
| Per tertile increase | 1.236 (1.122, 1.361) | < 0.001 |

Abbreviations: *OR*, Odd ratios; *CI*, confidence interval; T1, First tertile; T2, Second tertile; T3, Third tertile; AIP index, Atherogenic Index of Plasma; TyG index, triglyceride glucose index; METS-IR, metabolic score for Insulin resistance; ln (TG/HDL-C), natural logarithm of the triglyceride to high-density lipoprotein cholesterol ratio. Models were adjusted for demographic characteristics (age, gender), BMI, medical history (hypertension, hyperlipidemia) and behavioural risk factors (drinking, smoking, physical activity).

*P* trend: calculated according to tertile order rank.

Supplementary Table 8. Logistics regression analysis was conducted to assess the relationship between alternative indexes of insulin resistance and coronary heart disease excluding participants with diabetes.

| Alternative indexes  of insulin resistance | *OR (95%CI)* | | |
| --- | --- | --- | --- |
|  | Model 1 | Model 2 | Model 3 |
| **AIP index** |  |  |  |
| Per one-unit increase | 1.519(1.253, 1.842) | 1.533(1.253, 1.876) | 1.504(1.225, 1.847) |
| Tertile |  |  |  |
| T1 | Ref. | Ref. | Ref. |
| T2 | 1.306 (1.080, 1.580) | 1.249 (1.026, 1.520) | 1.252 (1.027, 1.527) |
| T3 | 1.554 (1.269, 1.904) | 1.520 (1.232, 1.873) | 1.507 (1.219, 1.863) |
| *P*_trend_ | < 0.001 | < 0.001 | < 0.001 |
| Per tertile increase | 1.236 (1.119, 1.365) | 1.242 (1.115, 1.382) | 1.230 (1.108, 1.365) |
| **TyG index** |  |  |  |
| Per one-unit increase | 0.973(0.879, 1.077) | 1.058(0.9515, 1.176) | 1.071(0.9603, 1.193) |
| Tertile |  |  |  |
| T1 | Ref. | Ref. | Ref. |
| T2 | 1.042 (0.904, 1.201) | 1.107 (0.955, 1.283) | 1.111 (0.957, 1.289) |
| T3 | 0.860 (0.685, 1.081) | 1.017 (0.805, 1.284) | 1.047 (0.826, 1.326) |
| *P*_trend_ | 0.420 | 0.546 | 0.423 |
| Per tertile increase | 0.968 (0.856, 1.094) | 1.069 (0.942, 1.213) | 1.084 (0.952, 1.233) |
| **METS-IR** |  |  |  |
| Per one-unit increase | 1.028(1.020, 1.036) | 1.026(1.018, 1.035) | 1.033(1.023, 1.044) |
| Tertile |  |  |  |
| T1 | Ref. | Ref. | Ref. |
| T2 | 1.488 (1.306, 1.695) | 1.437 (1.255, 1.644) | 1.425 (1.244, 1.633) |
| T3 | 1.550 (1.277, 1.880) | 1.531 (1.252, 1.871) | 1.602 (1.257, 2.042) |
| *P*_trend_ | < 0.001 | < 0.001 | < 0.001 |
| Per tertile increase | 1.444 (1.297, 1.607) | 1.419 (1.272, 1.584) | 1.557 (1.369, 1.772) |
| **ln (TG/HDL-C)** |  |  |  |
| Per one-unit increase | 1.199(1.103, 1.304) | 1.204(1.103, 1.314) | 1.194(1.092, 1.305) |
| Tertile |  |  |  |
| T1 | Ref. | Ref. | Ref. |
| T2 | 1.306 (1.080, 1.579) | 1.249 (1.026, 1.521) | 1.252 (1.027, 1.527) |
| T3 | 1.554 (1.272, 1.899) | 1.520 (1.234, 1.872) | 1.506 (1.214, 1.869) |
| *P*_trend_ | < 0.001 | < 0.001 | < 0.001 |
| Per tertile increase | 1.236 (1.119, 1.365) | 1.242 (1.115, 1.382) | 1.230 (1.108, 1.365) |

Abbreviations: *OR*, Odd ratios; *CI*, confidence interval; T1, First tertile; T2, Second tertile; T3, Third tertile; AIP, Atherogenic Index of Plasma; TyG index, triglyceride glucose index; METS-IR, metabolic score for Insulin resistance; ln (TG/HDL-C), logarithm of the triglyceride to high-density lipoprotein cholesterol ratio.

Model 1: adjusted for none;

Model 2: general demographic characteristics (age, gender) were adjusted;

Model 3: adjusted for BMI, medical history (hypertension, hyperlipidemia) and behavioural risk factors (drinking, smoking, physical activity) on the basis of model 2;

*P* trend: calculated according to tertile order rank.


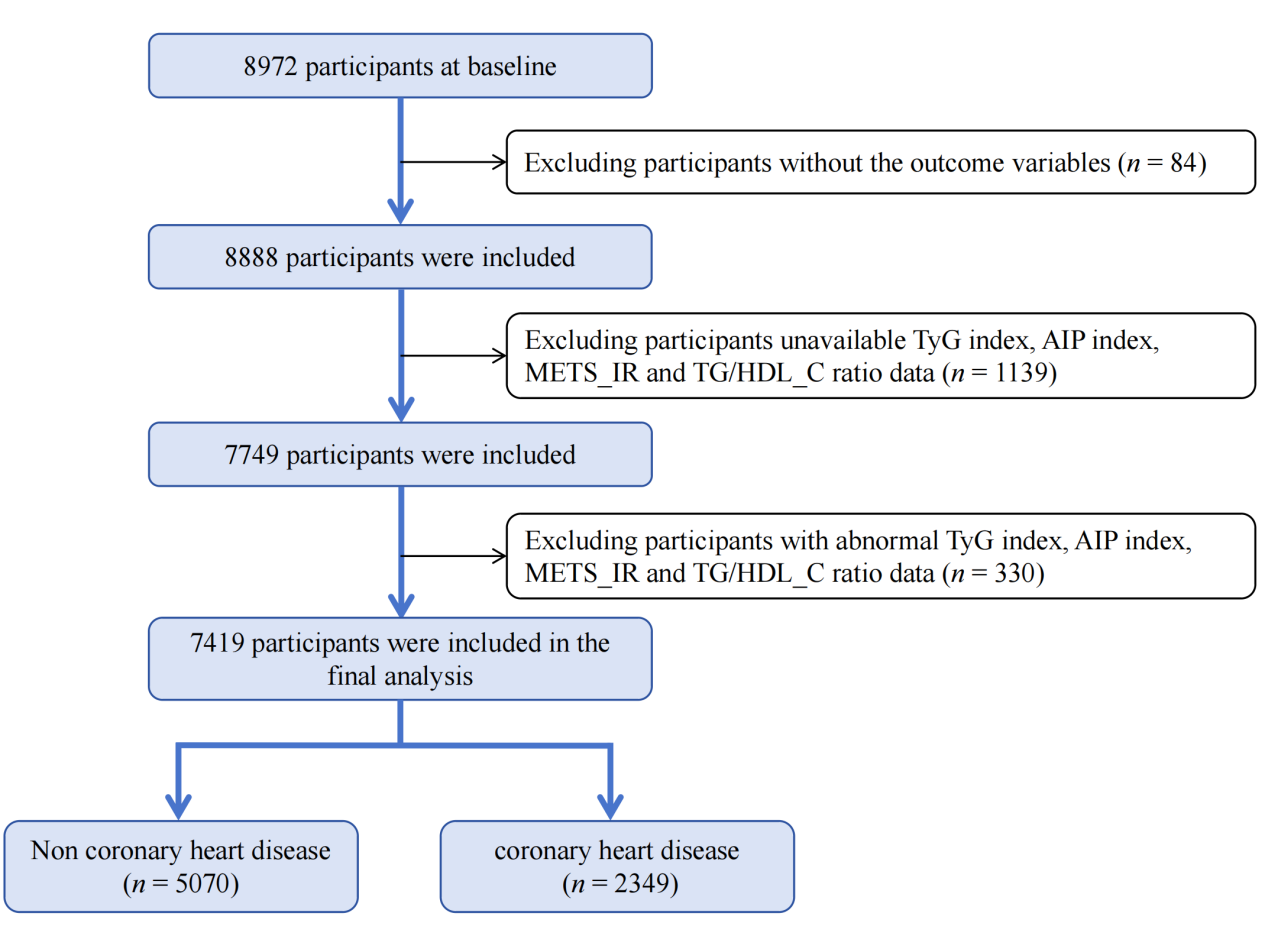


Supplementary Figure 1. Flowchart of participants recruitment and selection.


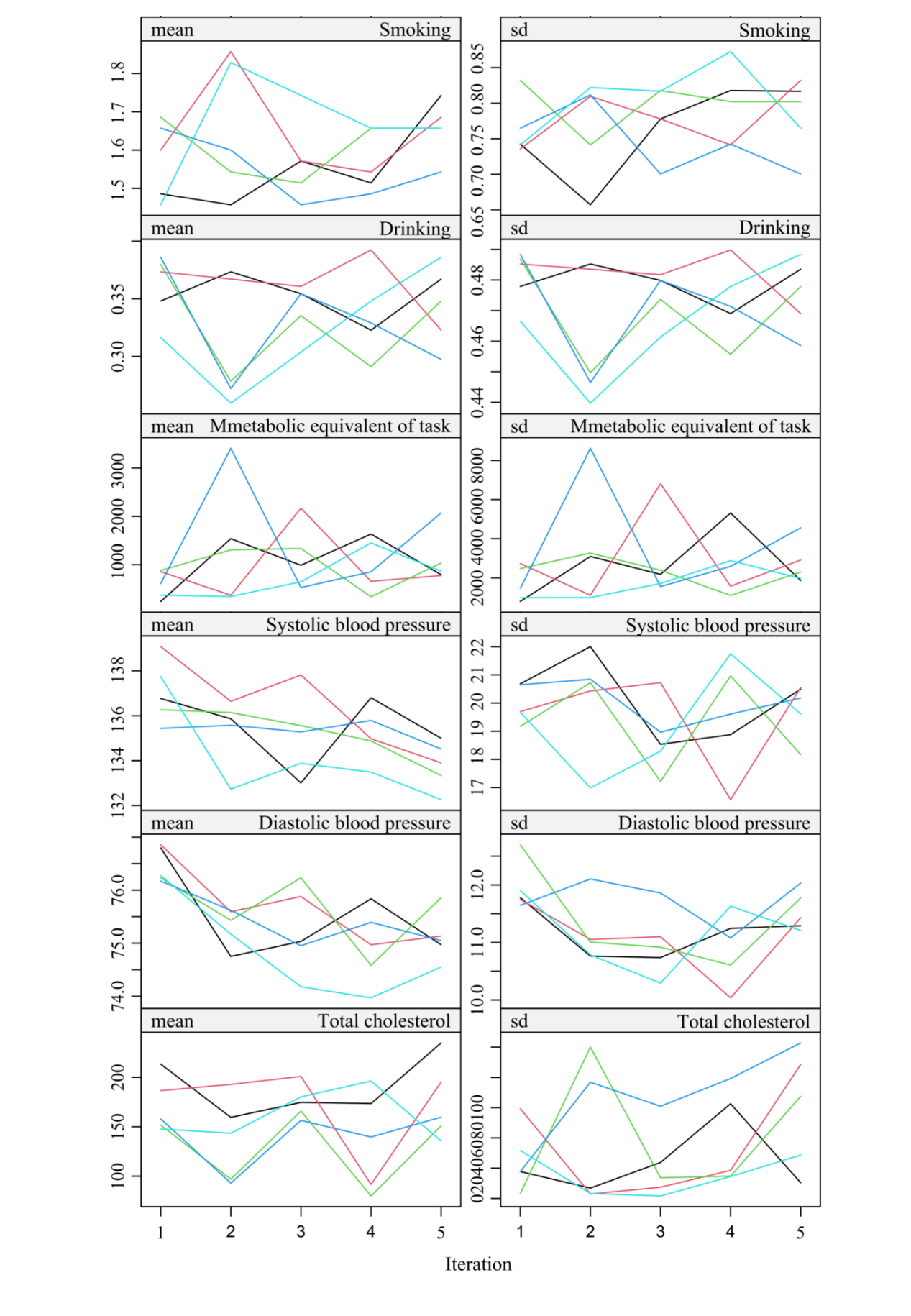


Supplementary Figure 2. Trace plots for key covariates in the multiple imputation procedure. Trace plots of chain-specific means (left column) and standard deviations (right column) for smoking, drinking, metabolic equivalent of task, systolic blood pressure, diastolic blood pressure and total cholesterol across five iterations of the MICE algorithm. Each coloured line represents one of the five imputed datasets. The chains show good mixing and random fluctuations around stable values without systematic upward or downward trends, indicating satisfactory convergence of the multiple imputation model for these covariates.


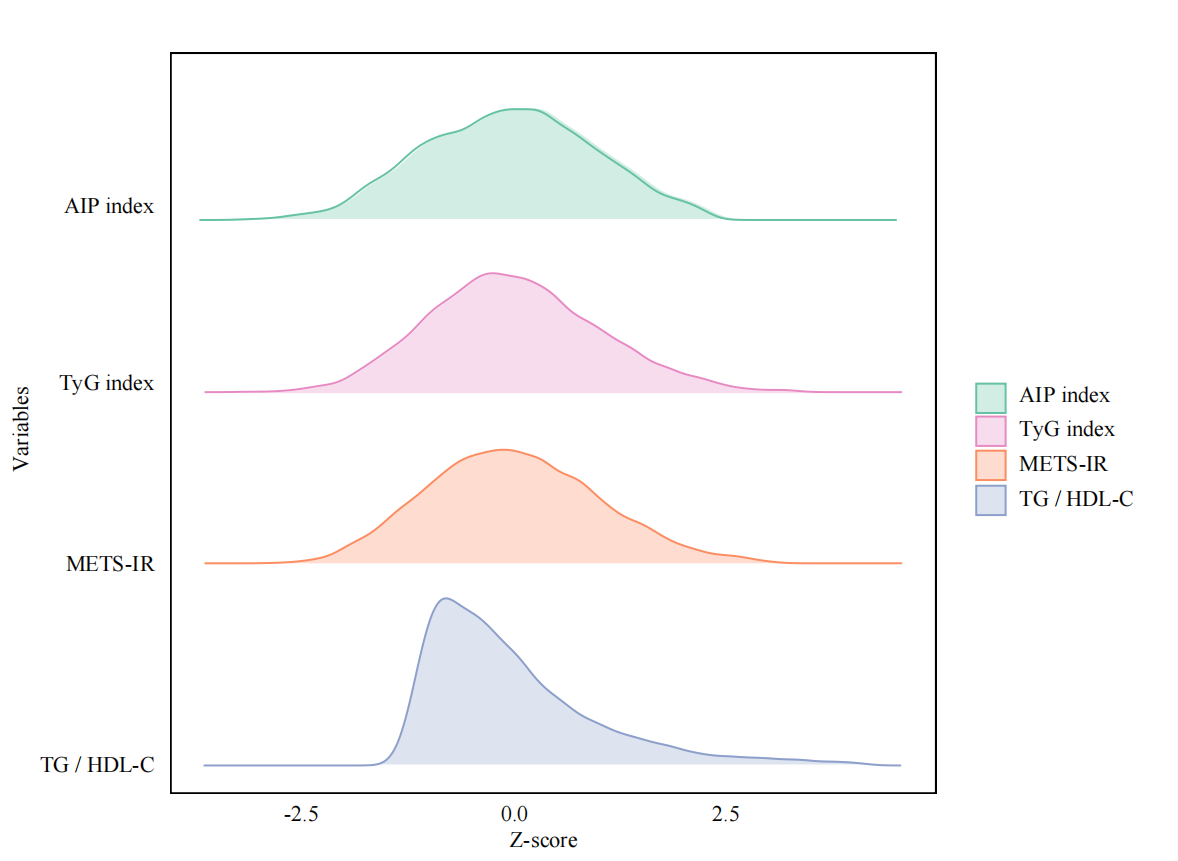
Supplementary Figure 3. Kernel density distributions of four standardized alternative indices of insulin resistance (AIP index, TyG, index METS-IR, and TG/HDL-C). Curves represent probability density. AIP index and TyG index appear approximately symmetric and near-normal; METS-IR shows mild right-skew; TG/HDL-C displays pronounced right-skew with a long right tail. All variables were Z-score standardized to compare distributional shape across indices.


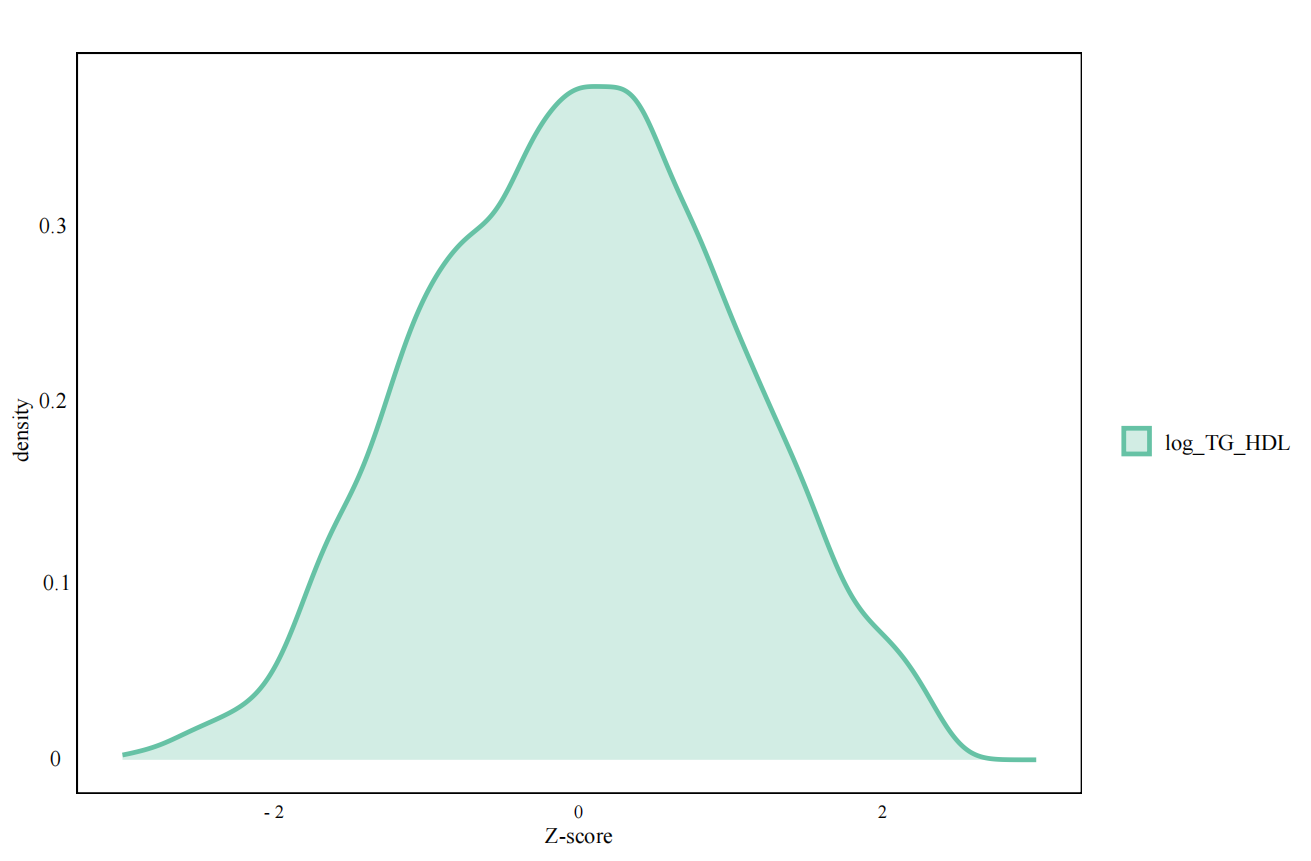


Supplementary Figure 4. Distribution of standardized ln (TG/HDL-C) using kernel density estimation.


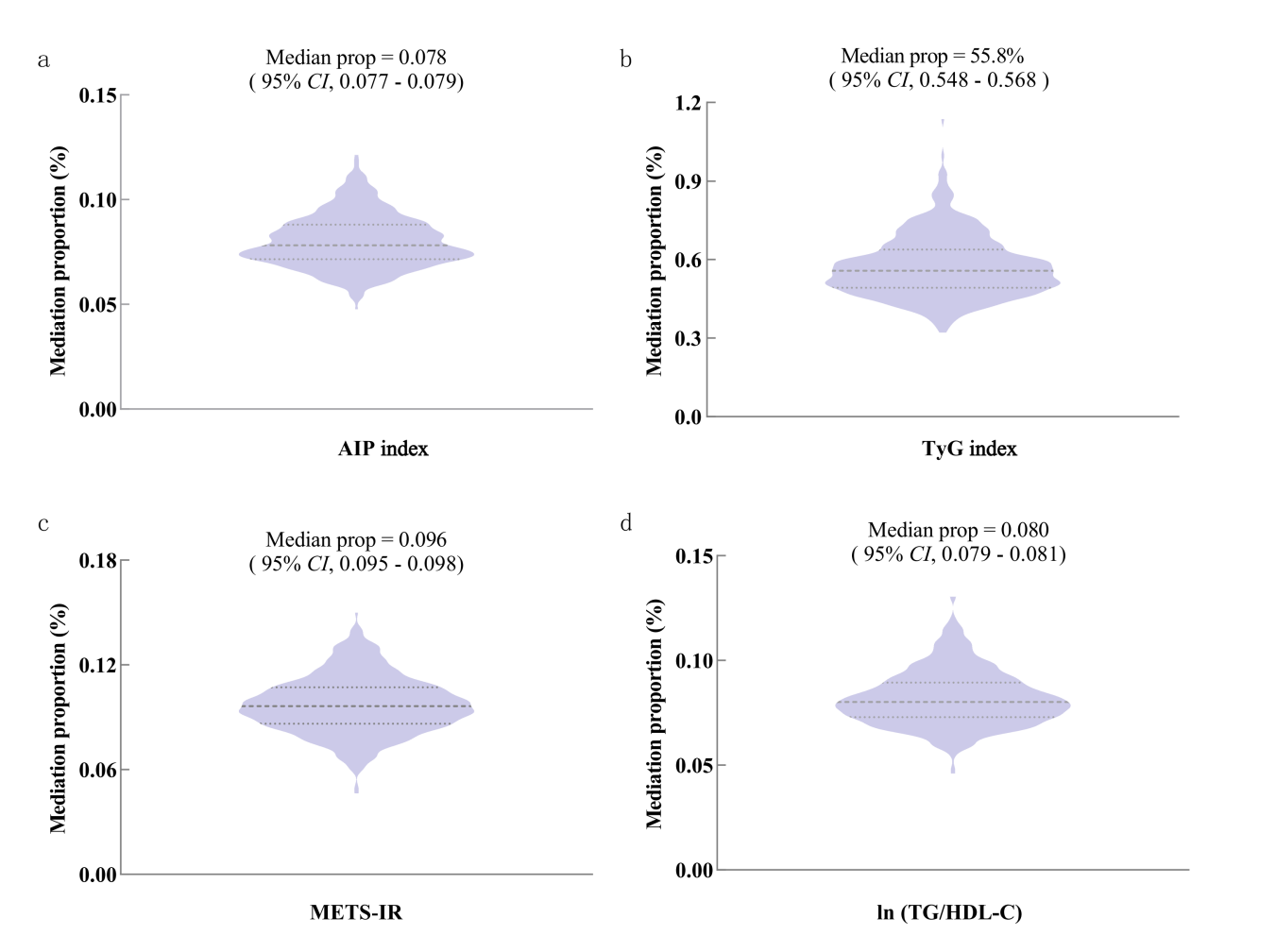


Supplementary Figure 5. Probit-based sensitivity analysis demonstrating the robustness of the primary logit results. Probit-based sensitivity analysis was performed by randomly extracting 80% of samples from the dataset for causal mediation analysis (n = 500 times). The average direct effect (ADE) represents the portion of the total effect that is independent of the mediator. The average causal mediation effect (ACME) denotes the portion of the total effect that can be explained by the mediator. The mediation proportion (prop) was calculated by ACME/(ADE + ACME) × 100%. Probit analysis was employed in the causal mediation modelling to estimate ADE and ACME, expressed as β coefficients (slopes) from the probit regression. The violin plots display the distribution of the mediation proportion across 500 bootstrap iterations, with three dashed lines indicating the median (center), the 25th percentile (lower), and the 75th percentile (upper). The 95% *CI*s for the mediation proportions were calculated using the formula ± 1.57 × *IQR* √n. All statistical tests were two-sided, and *P* < 0.05 was considered statistically significant. The variable n indicates the number of participants included.
